# *MicroRNA-140* mediates RB tumor suppressor function to control stem cell-like activity through interleukin-6

**DOI:** 10.18632/oncotarget.14681

**Published:** 2017-01-16

**Authors:** Akiyo Yoshida, Shunsuke Kitajima, Fengkai Li, Chaoyang Cheng, Yujiro Takegami, Susumu Kohno, Yuan Song Wan, Naoyuki Hayashi, Hayato Muranaka, Yuuki Nishimoto, Naoko Nagatani, Takumi Nishiuchi, Tran C Thai, Sawako Suzuki, Shinji Nakao, Tomoaki Tanaka, Osamu Hirose, David A. Barbie, Chiaki Takahashi

**Affiliations:** ^1^ Division of Oncology and Molecular Biology, Cancer Research Institute, Kanazawa University, Kanazawa, Ishikawa 920-1192, Japan; ^2^ Deperment of Cellular Transplantation Biology, Graduate School of Medical Science, Kanazawa University, Kanazawa, Ishikawa 920-8641, Japan; ^3^ Department of Medical Oncology, Dana-Farber Cancer Institute, Boston, MA02215, USA; ^4^ DNAFORM Precision Gene Technologies, Yokohama, Kanagawa, 230-0046, Japan; ^5^ Department of Health and Nutrition, Faculty of Human Health Science, Kanazawa Gakuin University, Kanazawa, Ishikawa, 920-1302, Japan; ^6^ Division of Functional Genomics, Advanced Science Research Center, Kanazawa University, Kanazawa, Ishikawa 920-0934, Japan; ^7^ Deperment of Clinical Cell Biology and Medicine, Graduate School of Medicine, Chiba University, Chiba, Chiba 260-8670 Japan; ^8^ Division of Electrical Engineering and Computer Science, Graduate School of Natural Science and Technology, Kanazawa University, Kanazawa, Ishikawa 920-1192, Japan

**Keywords:** cancer, cancer stem cells, RB, mir-140, interleukin-6

## Abstract

We established an *in vitro* cell culture system to determine novel activities of the retinoblastoma (Rb) protein during tumor progression. Rb depletion in *p53*-null mouse-derived soft tissue sarcoma cells induced a spherogenic phenotype. Cells retrieved from Rb-depleted spheres exhibited slower proliferation and less efficient BrdU incorporation, however, much higher spherogenic activity and aggressive behavior. We discovered six miRNAs, including mmu-miR-18a, -25, -29b, -140, -337, and -1839, whose expression levels correlated tightly with the Rb status and spherogenic activity. Among these, mmu-miR-140 appeared to be positively controlled by Rb and to antagonize the effect of Rb depletion on spherogenesis and tumorigenesis. Furthermore, among genes potentially targeted by mmu-miR-140, Il-6 was upregulated by Rb depletion and downregulated by *mmu-mir-140* overexpression. Altogether, we demonstrate the possibility that *mmu-mir-140* mediates the Rb function to downregulate Il-6 by targeting its 3′-untranslated region. Finally, we detected the same relationship among RB, hsa-miR-140 and IL-6 in a human breast cancer cell line MCF-7. Because IL-6 is a critical modulator of malignant features of cancer cells and the RB pathway is impaired in the majority of cancers, hsa-miR-140 might be a promising therapeutic tool that disrupts linkage between tumor suppressor inactivation and pro-inflammatory cytokine response.

## INTRODUCTION

MicroRNAs (miRNAs) are non-coding 18 to 25 base pair ribonucleotides that post-transcriptionally downregulate proteins by binding to a complementary sequence in the 3′-untranslated region (3′UTR) of target gene mRNA [1, 2]. Recently, miRNAs have emerged as critical modifiers of malignant phenotypes in several cancers [3–5]. Extensive downregulation of miRNAs was observed in human tumor cells relative to normal tissues [6, 7]. Lower expression of components mediating miRNA biosynthesis such as DROSHA and DICER1 was correlated with poor prognosis in ovarian cancers and neuroblastomas [8, 9]. These findings indicate tumor-suppressive functions that are shared by a wide variety of miRNAs. Indeed, *Dicer1* knockout mice, in which miRNA expression is globally repressed, exhibit a tumor-prone phenotype [10, 11], even though some miRNAs such as *miR-21* have oncogenic functions [12].

The tumor-suppressive functions of miRNAs are attained primarily by their ability to interfere with the translation of oncogenic mRNAs. For example, the *miR-200* family antagonizes the epithelial-mesenchymal transition (EMT) associated with cancer metastasis by downregulating transcriptional suppressors of E-cadherin, such as *ZEB1* and *ZEB2* [13]. Furthermore, *let-7*, *miR-15a, miR-31, miR-34, miR-205* and others were demonstrated to suppress Ras, Myc, Bcl2, Notch, E2F1 or CyclinD1 [14], suggesting a tumor-suppressive activity for these miRNAs. However, exactly how these tumor-suppressive miRNAs are regulated during tumor progression remains poorly defined.

The tumor suppressor protein retinoblastoma (RB) is genetically or functionally inactivated in many human cancers, and exerts its tumor-suppressive functions through physical interactions with various effector molecules including E2F transcription factors, tissue-specific transcription factors, LxCxE motif-containing chromatin modifiers, and the E3 ubiquitin protein ligase SKP2. Due to the large variation in its binding partners, the RB transcriptional complex can either promote or repress expression of its target genes [15].

In this study, we employed an *in vitro* model of cancer progression wherein Rb inactivation enhances stem cell-like activities. We identified miRNAs whose levels differ in close association with the RB status and stem cell-like activities. This unveiled a relationship between RB and miR-140; depletion of RB downregulates miR-140. The *mir-140* has been implicated in the suppression of hepatocellular carcinoma, non-small cell lung cancer, colon cancer, breast cancer and ovarian cancer through the inhibition of growth factor signaling [16–20]. We further identified IL-6 gene as a possible direct target of *mir-140*. Rb depletion indeed upregulated IL-6 expression, which was antagonized by overexpression of *mir-140*. Therefore, this study proposes that Rb downregulates IL-6 through upregulation of *mir-140*.

## RESULTS

### Il-6 mRNA upregulation is associated with an undifferentiated state following Rb inactivation

**Figure 1:**
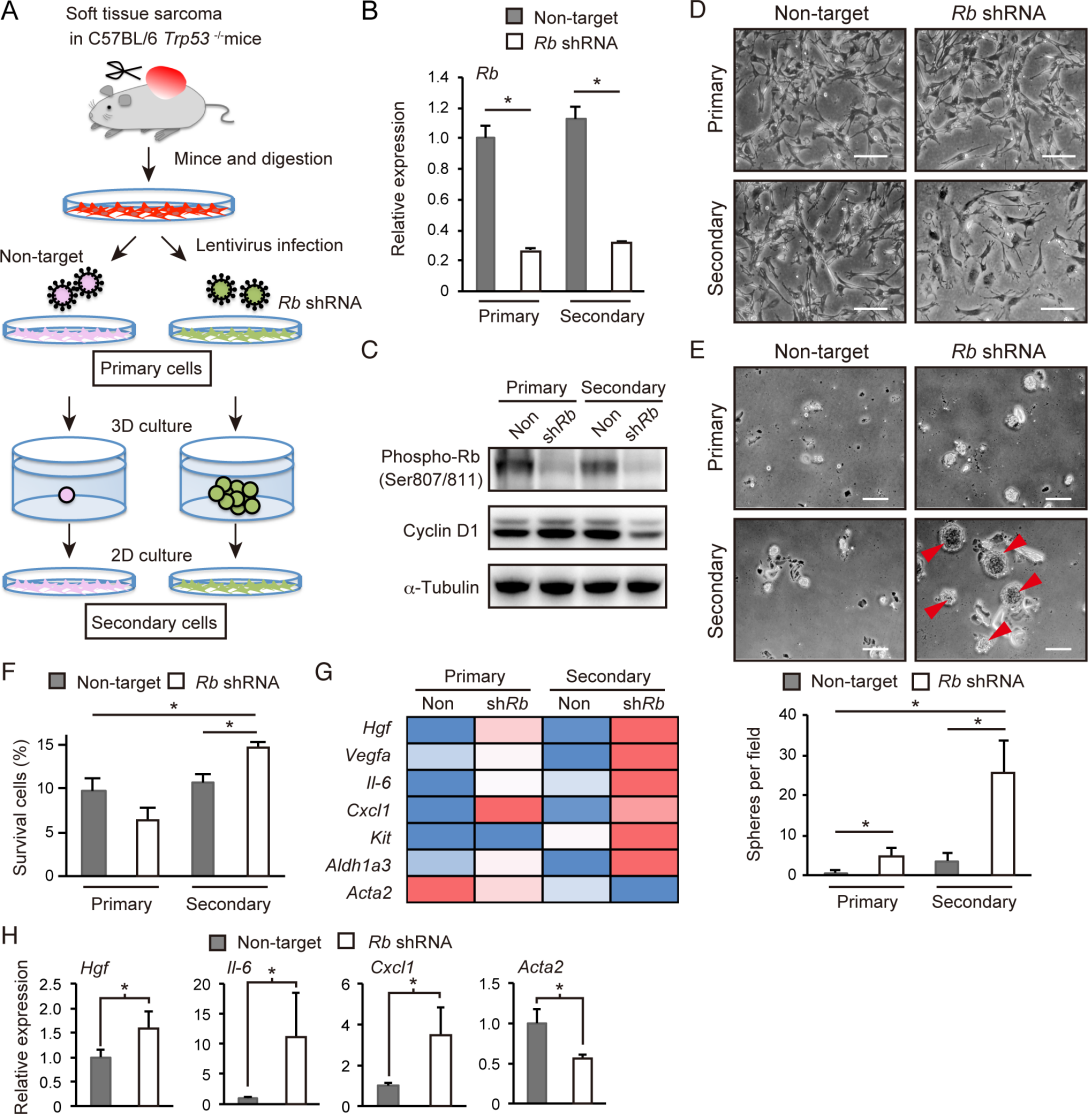
Analysis of Rb depletion-induced spherogenic cells (**A**) Protocol for the preparation of Rb depletion-induced spherogenic cells (Rb-depleted secondary cells) from *p*53-null soft tissue sarcoma cells. (**B**) RT-qPCR of Rb in *p*53-null sarcoma cells transduced with the indicated vector. *N* = 3. Columns represent the mean and standard deviation (S.D.) unless otherwise indicated. (**C**) Immunoblot (IB) of the indicated proteins in *p*53-null soft tissue sarcoma cells transduced with the indicated vector. (**D**) Phase-contrast images of *p*53-null soft tissue sarcoma cells grown in monolayer culture transduced with the indicated vector. Scale bar: 200 μm. (**E**) Spheres (arrowheads) derived from *p*53-null soft tissue sarcoma cells transduced with the indicated vector (upper). Scale bar: 200 μm. Sphere numbers per field (lower). *N* = 3. (**F**) Cell survival rates for *p*53-null soft tissue sarcoma cells transduced with the indicated vector after treatment with 250 nM doxorubicin for 72 hours. *N* = 3. (**G**) Heat map of rpkm values of the indicated genes in *p*53-null soft tissue sarcoma cells transduced with the indicated vector. (**H**) 1 × 10^5^ cells transduced with the indicated vector were injected into C57BL/6 mice. RT-qPCR results of the indicated genes in tumors derived from implanted *p*53-null soft tissue sarcoma cells transduced with the indicated shRNA are shown (day 24). *N* = 3.

Previously, we demonstrated that a *p53*-null background facilitates cells to acquire undifferentiated phenotypes including increased self-renewal upon RB inactivation [21, 22]. To determine the RB-inactivation signature in the context of gaining self-renewal activity in a *p53*-null genetic background, we employed a soft tissue sarcoma model. *p53*^−/−^ mice in a C57BL/6 background developed soft tissue-derived sarcoma with a frequency of approximately 20%. Cells derived from these tumors were readily adaptable to 2-dimensional (2D) culture, and appeared to be poorly spherogenic when cultivated in serum-free medium supplemented with EGF, bFGF and B-27 in a non-adherent dish type. However, upon Rb depletion, these cells efficiently formed small spheres up to day 14 in sphere culture (Figure 1A–1E), suggesting enhanced self-renewal activity in Rb depleted cells.

Next we recovered viable cells from the spheres derived from Rb-depleted cells. Spheres with surface areas > 5,000 μm^2^ and ratios of the longest/shortest diameter (L/S ratio) < 1.5 were manually picked, disaggregated with a cell strainer, and plated onto a 2D culture dish (Figure 1A and Supplementary Figure 1A). We referred to these cells as “Rb-depleted secondary cells” and conducted all analysis of these cells before they reached passage 4. “Control secondary cells” were derived from cells surviving 3D culture conditions for sphere formation, although these cells did not form visible spheres.

Both control and Rb-depleted secondary cells re-adapted well to 2D culture conditions. However, as compared to control and other primary cells, Rb-depleted secondary cells exhibited flattened cell shapes and slower growth (Figure 1D). In agreement, Rb-depleted secondary cells showed decreased cyclin D1 expression (Figure 1C) and less efficient BrdU incorporation (Supplementary Figure 1B). However, Rb-depleted, sphere-derived secondary cells exhibited the highest spherogenic activity, resistance to doxorubicin treatment and metastatic activity in mice when compared to the three other types of cells (Figure 1E and 1F, and Supplementary Figure 1C).

We analyzed all four types of cells by RNA sequencing. We found that the well-established cancer stem cell marker *Aldh1a3* was highly expressed in Rb-depleted secondary cells, strongly implicating the enrichment of stem cell-like cells in this cell population (Figure 1G). In addition to *Aldh1a3*, we found that *Hgf*, *Vegfa*, *Il-6*, *Cxcl1* and *Kit* were highly expressed in Rb-depleted secondary cells (Figure 1G). Of note, except for *Kit*, all these genes were upregulated in Rb-depleted primary cells as well, implicating a genetic interaction between Rb and these genes. Additionally, we observed that Rb depletion suppressed *smooth muscle actin 2* (*Acta2*) expression (Figure 1G). Subcutaneous tumors derived from Rb-depleted cells transplanted into C57BL/6 mice also showed increased *Hgf*, *Il-6*, and *Cxcl1* expression and decreased *Acta2* expression (Figure 1H). Collectively, these findings indicate that Rb depletion induces upregulation of specific growth factors and cytokines, and dedifferentiation in **p*53*-null soft tissue sarcoma cells.

### miRNA expression profiling identifies mmu-miR-140 downregulation in conjunction with this Rb-inactivated undifferentiated state

**Figure 2:**
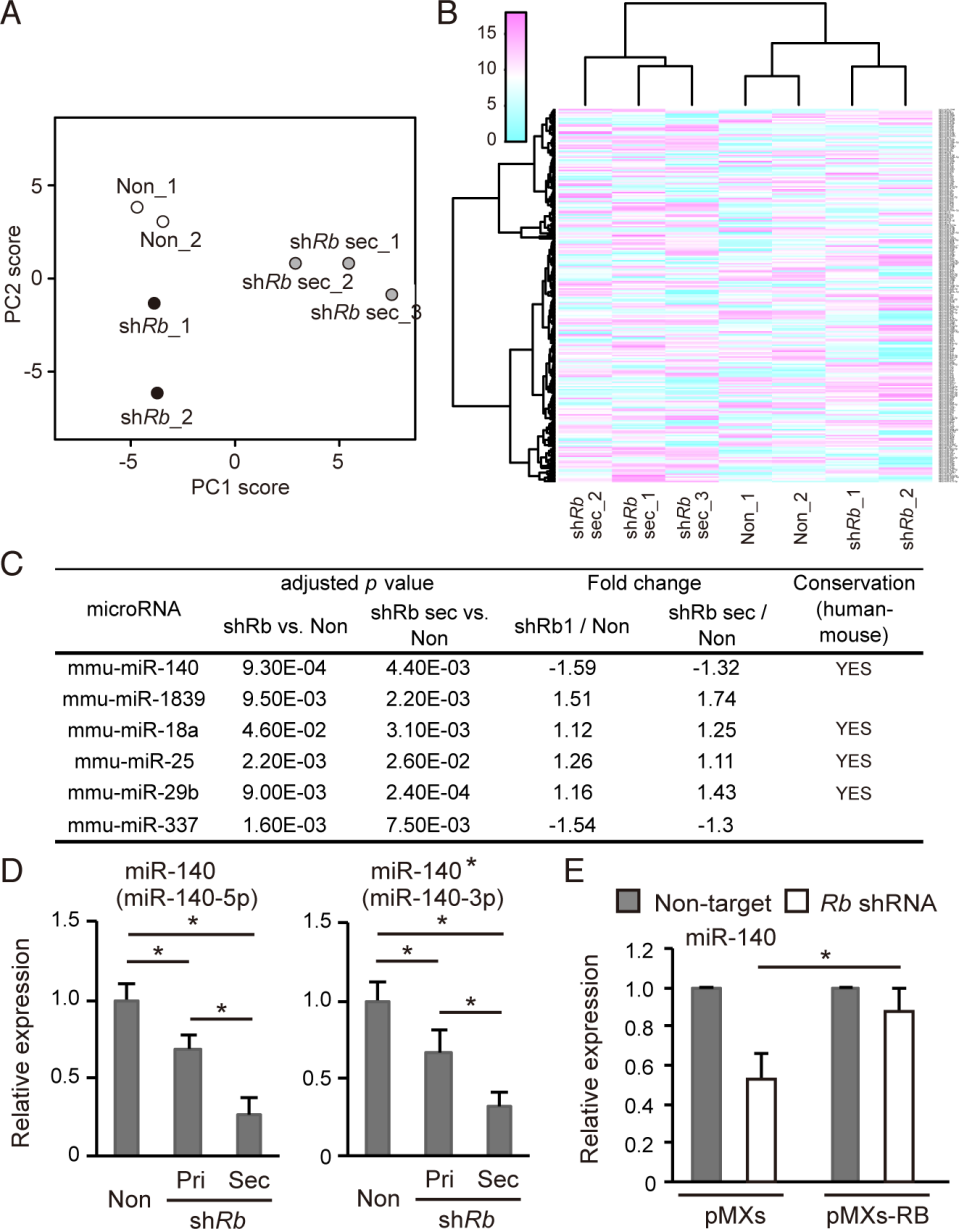
Rb depletion downregulates *mir-140* expression (**A**) Principal components analysis of normalized array data for 252 microRNAs. The scores of the first two principal components are shown. The proportions of variance for PC1 and PC2 were 54.4% and 23.0% respectively. (Cumulative proportion of PC1 and PC2 is 77.4%). (**B**) Unsupervised hierarchical clustering of miRNA data for the 252 normalized miRNAs. Each column represents a sample, and each row represents the expression level of a miRNA. (**C**) The list of miRNAs differentially expressed depending on the Rb depletion status and the culture condition. The expression data of miRNA were analyzed by one-way ANOVA, and then by Dunnett's post-hoc test, and compared to control cells. Homology denotes the presence of homologous sequence in *Homo sapiens* and *Mus musculus*. (See Materials and Methods). (**D**) RT-qPCR of miR-140 (left) and miR-140* (right) in *p*53-null soft tissue sarcoma cells transduced with the indicated vector. *N* = 3. (**E**) RT-qPCR of miR-140 in *p*53-null soft tissue sarcoma cells transduced with the indicated vector. *N* = 3.

We next determined the Rb-inactivation signature of miRNA in addition to mRNA. To this end, we performed a miRNA array assay with control primary cells and Rb-depleted primary and secondary cells. We conducted principal component analysis (PCA) using a variance-covariance matrix. The cumulative contribution rate of the first (PC1) and second principal components (PC2) was 77%. The samples were divided into primary and secondary cells by the axis of the PC1 score, and control and Rb-depleted cells by the axis of the PC2 score (Figure 2A). Then we conducted an unsupervised hierarchical clustering analysis using Ward's method. The samples were successfully divided into clusters of primary and secondary cells. The primary cell clusters were also subdivided into clusters of control and Rb-depleted cells (Figure 2B). Based on these results, we concluded that the obtained miRNA expression profiles were well organized and specific in each cell type.

Using these miRNA expression profiles, we identified miRNAs whose expression level correlate with Rb status and stem cell-like features simultaneously. At first, we conducted one-way analysis of variance (ANOVA) with 252 miRNAs. The resultant 37 miRNAs with adjusted *p* values < 0.1 were subjected to Dunnett's test using non-target cells as control, and we then identified six miRNAs (mmu-miR-18a, -25, -29b, -140, -337, and -1839) with adjusted *p* values < 0.05 (Figure 2C). Among these six miRNAs, mmu-miR-140 and -337 appeared to be downregulated by Rb deletion in both 2D-cultured and sphere-derived cells. Of these two miRNAs, mmu-miR-140 exhibited more robust fold change. In addition, mmu-miR-140 sequence was conserved between mouse and human (Figure 2C). For these reasons, our subsequent studies focused on mmu-miR-140.

We validated the expression of mmu-miR- 140-5p (miR-140) and mmu-miR-140-3p (miR-140*) in the three cell types by reverse transcription-quantitative PCR (RT-qPCR) (Figure 2D and Supplementary Figure 2A). These results indicated that both forms of *mmu-mir-140* are downregulated by Rb depletion in primary cells; this downregulation was more robust in Rb-depleted secondary cells (Figure 2D). In addition, we demonstrated that mmu-miR-140 downregulation induced by Rb depletion was antagonized by RB overexpression (Figure 2E), suggesting that Rb upregulates mmu-miR-140 expression.

### *mmu-mir-140* antagonizes malignant features induced by Rb depletion

**Figure 3:**
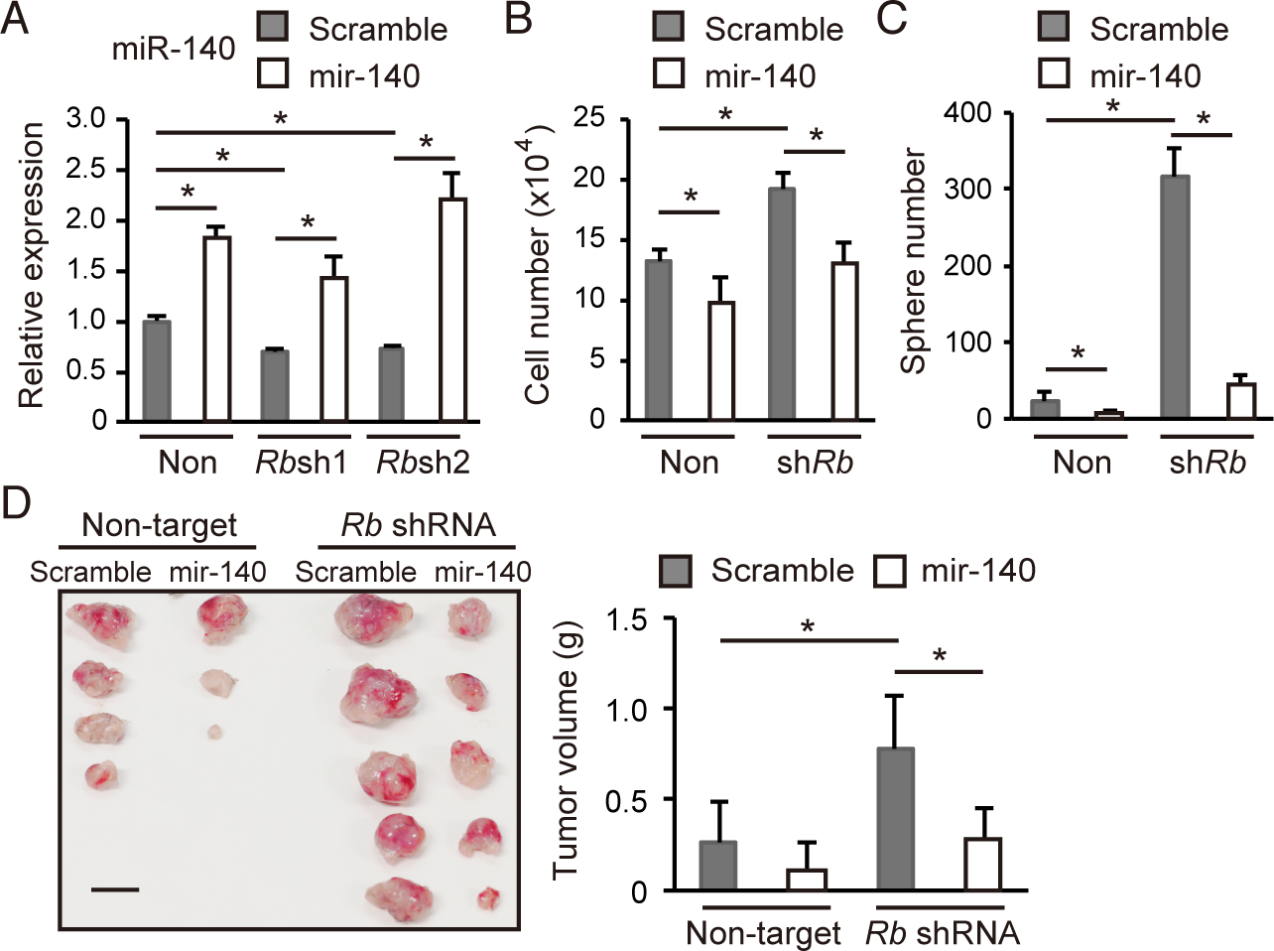
*mmu-mir-140* antagonizes the malignant features induced by Rb depletion (**A**) RT-qPCR of mmu-miR-140 in *p*53-null sarcoma cells transduced with the indicated vector. *N* = 3. (**B**) Total cell numbers of *p*53-null sarcoma cells transduced with the indicated vector after 72 hours culture. *N* = 3. (**C**) The number of spheres derived from 5 × 10^4^ of *p*53-null soft tissue sarcoma cells transduced with the indicated vector. *N* = 3. (**D**) Tumor initiation by 1 × 10^5^ cells transduced with the indicated vector in C57BL/6 mice (left; day 24). Scale bar: 10 mm. Tumors were weighed (right). *N* = 5.

Since *hsa-mir-140*, the human orthologue of *mmu-mir-140*, has been implicated in tumor suppression [16–20], we investigated whether *mmu-mir-140* mediates Rb function to suppress tumor development in the soft tissue sarcoma model. We transduced a lentiviral vector expressing *mmu-mir-140* (a precursor of mmu-miR-140 and mmu-miR-140*) or scrambled sequence into control or Rb-depleted cells. We verified mmu-miR-140 expression in transduced cells by RT-qPCR (Figure 3A). We found that *mmu-mir-14*0 overexpression did not induce apoptosis but attenuated the cell growth of both control and Rb depleted cells in 2D culture (Figure 3B and Supplementary Figure 3A and 3B). The degree of growth suppression was modest; however, *mmu-mir-140* overexpression significantly antagonized sphere formation induced by Rb depletion (Figure 3C). In addition, *mmu-mir-140* overexpression antagonized *in vivo* tumorigenicity enhanced by Rb depletion (Figure 3D). These findings indicate the possibility that *mmu-mir-140* mediates the function of Rb to suppress tumor development.

### Identification of genes regulated by Rb in a *mmu*-*mir*-*140*-dependent manner

**Figure 4:**
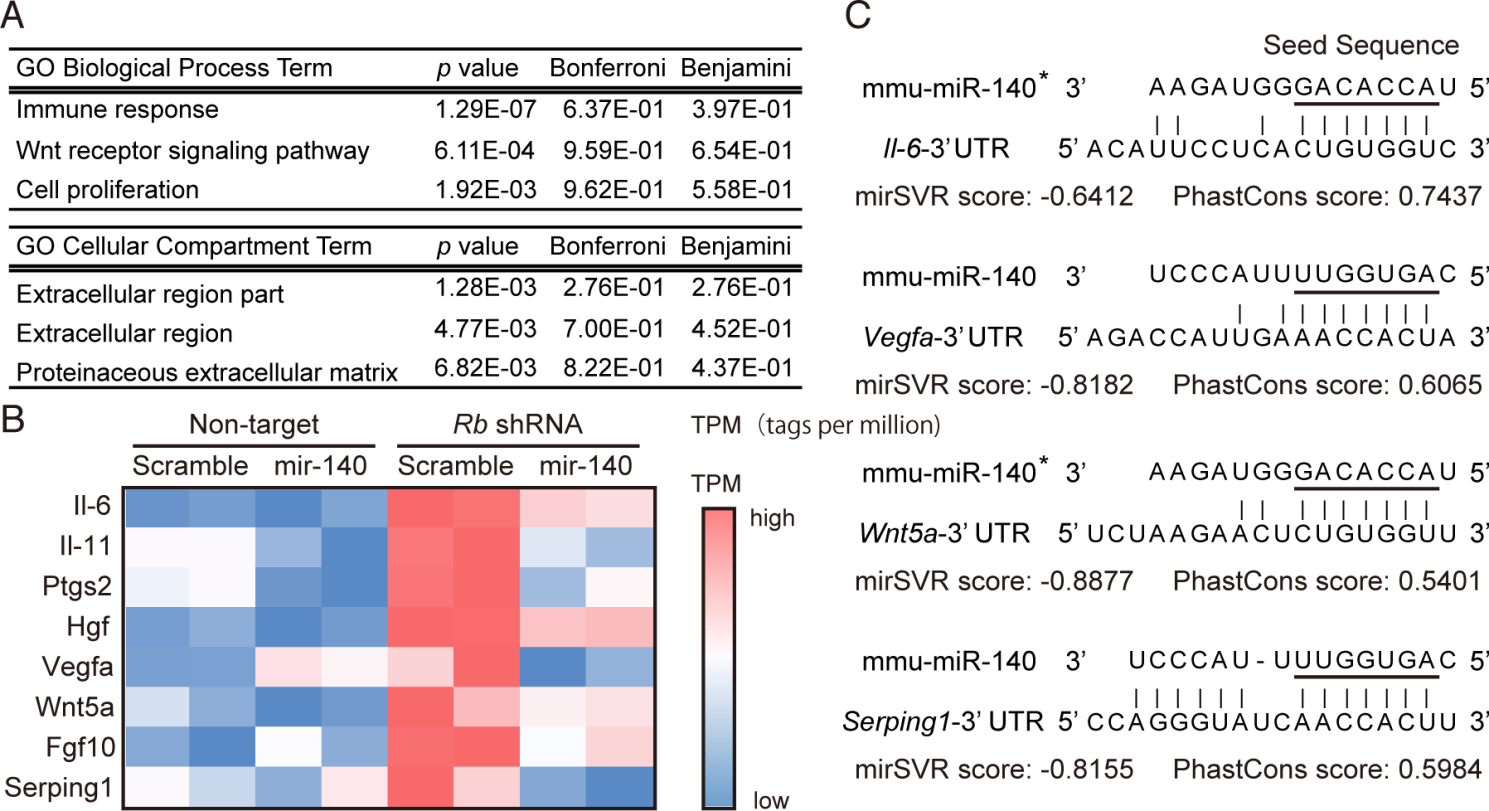
Identification of genes induced by Rb depletion in a *mir-140*-dependent manner (**A**) The ontology of the top 3 gene sets influenced by Rb depletion possibly in an *mir*-140-dependent manner was determined by GO Biological Process provided by DAVID. (**B**) A heat map illustrating the expression levels of the indicated genes in *p*53-null soft tissue sarcoma cells transduced with the indicated vector. (**C**) The sequences of mouse *Il-6, Vegfa, Wnt5a and Serping1* 3′UTR corresponding to the seed sequence of *mmu-mir-140* (underline) are presented with their mirSVR and PhastCons scores.

To further explore the possibility that *mir-140* may mediate the tumor-suppressive function of RB, we investigated genes regulated by Rb in a *mir-140*-dependent manner. To this end, we surveyed genes potentially targeted by *mmu-mir-140* among genes that were upregulated by Rb depletion. We searched for genes those predicted to be targeted by *mmu-mir-140* at microRNA.org. This search was made in the list of candidate genes identified by RNA-sequence as upregulated upon Rb depletion. We identified genes that had less than 0.5 mirSVR score to *mmu-mir-140* as candidate genes regulated by Rb in a *mir-140* dependent manner (Table 1). In addition, we performed transcriptome and gene ontology (GO)-enrichment analysis in Rb-depleted and/or *mmu-mir-140*-overexpressed soft tissue sarcoma cells by cap analysis gene expression (CAGE). The gene set induced by Rb depletion in a *mir-140*-dependent manner (induced by Rb depletion and suppressed by *mir-140* overexpression) was enriched by genes encoding soluble factors mediating immune responses and growth stimuli (Figure 4A, Supplementary Tables 1 and 2), such as *Il-6*, *Il-11*, *Hgf*, *Vegfa*, *Wnt5a*, *Fgf10* and *Serping1* (Figure 4B). The 3′UTR regions in these genes appeared to have highly possible *mir-140* target sequence (Figure 4C).

**Table 1:** Identification of the *mir-140*-target gene that contributes to Rb depletion-induced malignant phenotype

Gene	*a* value	*m* value	*q* value	rank	mirSVR score	
					miR140	miR140*
*Il6*	10.4	2.6	9.83E-26	1	−0.15	−0.64
*Itgb3*	10.5	1.6	1.60E-10	10	N/A	−0.91
*Igfbp5*	8.3	1.6	6.53E-03	191	−0.65	N/A
*Erlin2*	10.8	0.7	1.19E-02	232	−0.15	−0.90
*Vegfa*	13.0	0.6	1.52E-02	258	−0.82	N/A
*Lats2*	11.2	0.6	1.83E-02	281	−0.75	N/A
*Cd40*	2.3	3.1	2.60E-02	313	N/A	−0.63
*Serpind1*	7.4	1.5	2.65E-02	315	N/A	−0.99
*Slc2a1*	11.0	0.6	2.91E-02	330	−0.61	N/A

The genes listed here were upregulated by Rb depletion and possibly targeted by *mmu-mir-140*. These genes exhibited high mirSVR scores to miR-140 and/or miR-140*, and were sorted according to the *p* value of their rpkm values. mirSVR scores were obtained from microRNA.org [51, 52]. The *q* value represents adjusted *p* value by using the BH method, the *a* value represents the average expression level of triplicate samples, and the *m* value represents fold change on log2 scale.

### *mmu-mir-140* mediates Rb function to control *Il-6* expression

**Figure 5:**
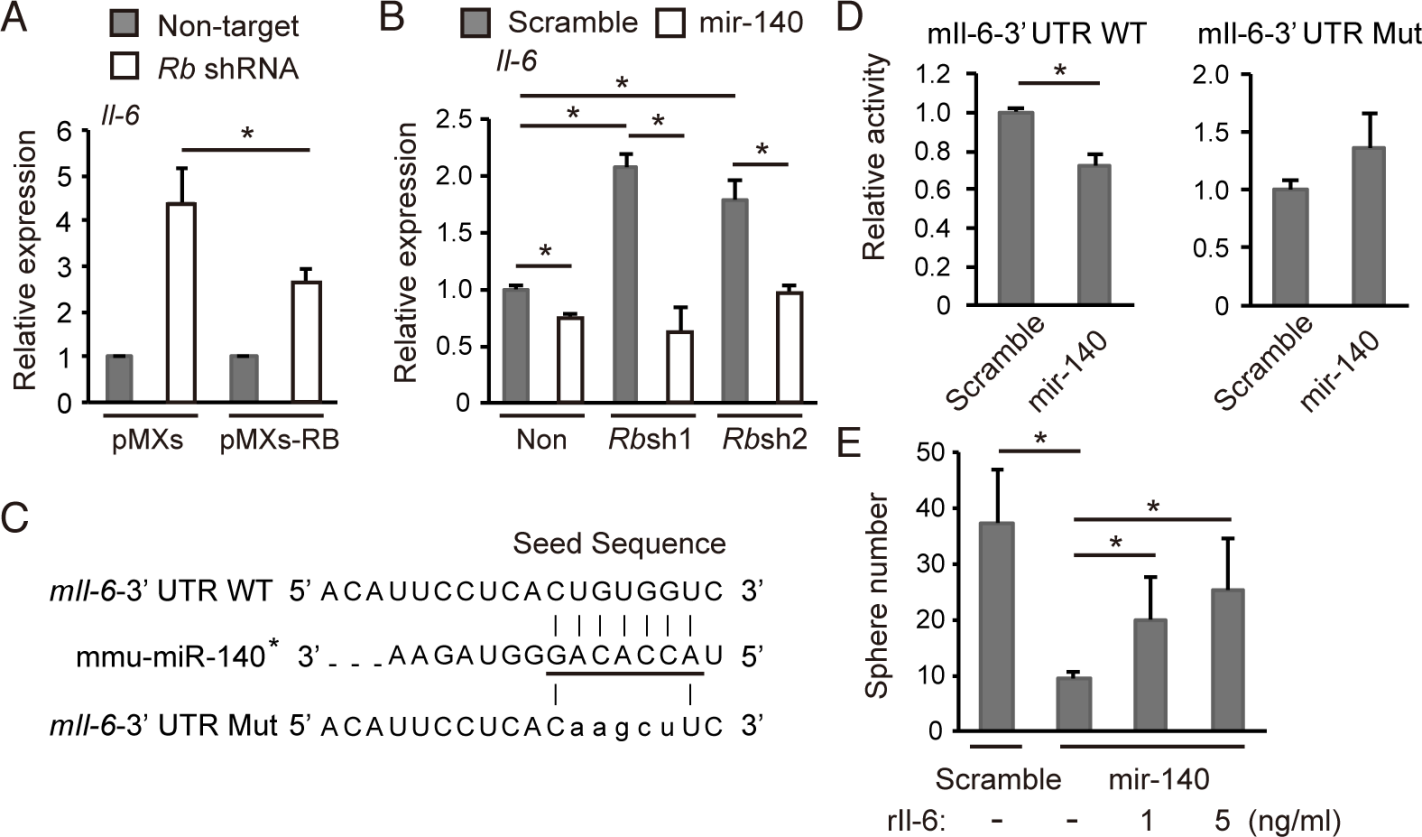
*mmu-mir-140* mediates Rb function to control *Il-6* expression (**A**) RT-qPCR of Il-6 in *p*53-null soft tissue sarcoma cells transduced with the indicated shRNA and retroviral vectors. *N* = 3. (**B**) RT-qPCR of Il-6 in *p*53-null soft tissue sarcoma cells transduced with the indicated vectors. *N* = 3. (**C**) The sequence of the mouse *Il-6 3′UTR* containing seed sequence of *mmu-mir-140* (underlined). The seed sequence of the mouse Il-6 3′UTR was mutated as shown (mIl-6-3′*UTR Mut*). (**D**) m*Il-6-3Times New RomanUTR WT* or m*Il-6-3′UTR Mut* was transduced into NIH3T3 cells together with the *mmu-mir-140* expression vector or control (scramble). After 48 hours, luciferase activity was measured. (**E**) Number of spheres derived from 5 × 10^4^ of *p*53-null soft tissue sarcoma cells transduced with the indicated vector in the presence of indicated concentration of recombinant mouse Il-6 (#406-ML-005, R&D Systems). *N* = 3.

We then focused on *Il-6* because it was the most upregulated gene following Rb depletion (Table 1). *Il-6* upregulation induced by Rb depletion was significantly antagonized by RB reconstitution (Figure 5A). In addition, *Il-6* has a *mir-140* target sequence in the 3′UTR of both the human and mouse gene. Indeed, *mmu-mir-140* overexpression significantly antagonized upregulation of *Il-6* abundance induced by Rb depletion (Figure 5B). On the other hand, *mmu-mir-140* depletion in Rb intact sarcoma cells was not sufficient to induce robust Il-6 upregulation or increased sphere forming activity (data not shown), suggesting that *mmu-mir-140* mediates Rb function to suppress Il-6 and sphere formation in a limited degree.

To determine whether the *Il-6* mRNA translation is directly suppressed by *mmu-mir-140*, we constructed luciferase reporter vectors carrying either a wild-type mouse *Il-6*-3′UTR (*mIl-6-3*′*UTR-WT*) or a mutated target sequence (*mIl-6-3*′*UTR-Mut*) (Figure 5C). We found that *mmu-mir-140* overexpression significantly repressed the activity of *mIl-6-3*′*UTR-WT* but not *mIl-6-3*′*UTR-Mut* (Figure 5D). Moreover, addition of recombinant mouse Il-6 significantly blocked the inhibition of spherogenesis caused by *mmu-mir-140* overexpression in a concentration-dependent manner (Figure 5E). These data implicate that *mmu-mir-140* suppresses IL-6 translation by binding to 3′UTR of *IL-6* mRNA, therefore may mediate Rb function to suppress tumor progression.

### *hsa*-*mir*-*140* suppressed *IL-6* upregulation following RB depletion in human breast cancer cells

**Figure 6:**
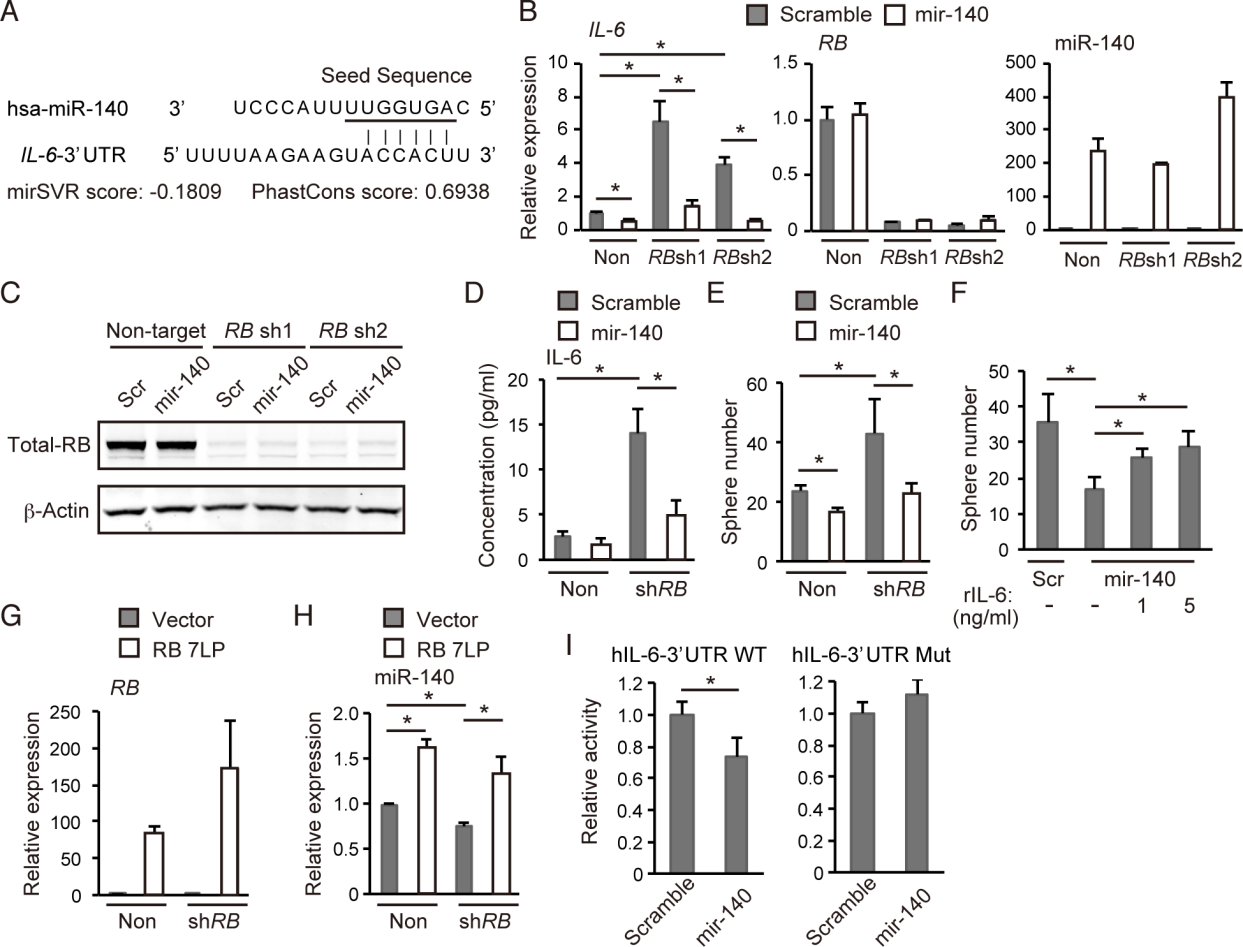
*hsa-mir-140* suppresses *IL-6* expression induced by RB depletion in human breast cancer cells (**A**) The sequence of the human *Il-6 3′UTR* and the seed sequence of *hsa-mir-140* (underlined) are indicated. mirSVR and Phantcons scores are indicated. (**B**) RT-qPCR of the indicated genes in MCF7 cells transduced with the indicated vector. *N* = 3. (**C**) IB of the indicated proteins in MCF-7 cells transduced with the indicated vector. (**D**) ELISA of human IL-6 levels in MCF-7 cells transduced with the indicated vector following 8 hours culture. (**E**) Number of spheres derived from 1.25 × 10^3^ of MCF-7 cells transduced with the indicated vectors. *N* = 3. (**F**) Number of spheres derived from 1.25 × 10^3^ of MCF-7 cells transduced with the indicated vector in the presence of indicated concentration of recombinant human IL-6 (#206-IL-010, R&D Systems). *N* = 3. (**G**) RT-qPCR of *RB7LP* in MCF-7 cells transduced with the indicated vector. *N* = 3. (**H**) RT-qPCR of hsa-miR-140 in MCF-7 cells transduced with the indicated vector. *N* = 3. (**I**) h*IL-6-3′UTR* WT or h*IL-6-3′UTR* Mut was transduced into MCF-7 cells together with the *hsa-mir-140* expression vector or control (scramble). After 48 hours, luciferase activity was measured.

To examine whether the *hsa-mir-140-IL-6* axis are involved in malignant phenotype induced by RB inactivation in human cancers, we employed MCF-7, a luminal-type breast cancer cell line. Since both RB and *hsa-mir-140* are frequently downregulated in basal-like breast cancer cells [20, 23], which typically show elevated cytokine secretion [24], we focused on human breast cancers. As mentioned above, the human *IL-6* gene has a *hsa-mir-140* target sequence in its 3′UTR (Figure 6A). We observed that the upregulation of *IL-6* abundance following RB-depletion in MCF-7 cells was significantly antagonized by *hsa-mir-140* overexpression (Figure 6B and 6C); this finding was confirmed by ELISA technique (Figure 6D). In addition, *hsa-mir-140* antagonized enhancement of sphere-forming activity induced by RB depletion (Figure 6E). Moreover, the addition of recombinant human IL-6 antagonized the inhibition of spherogenesis caused by *hsa-mir-140* overexpression in a concentration-dependent manner (Figure 6F).

On the other hand, while RB depletion in MCF-7 cells downregulated hsa-miR-140 expression, overexpression of a constitutively active form RB (RB7LP) [25] significantly upregulated hsa-miR-140 expression (Figure 6G and 6H). Furthermore, we constructed luciferase reporter vectors carrying either a wild-type human *IL-6*-3′UTR (*hIL-6-3′UTR-WT*) or a mutated target sequence (*hIL-6-3′UTR-Mut*) (Supplementary Figure 4A). We found that *hsa-mir-140* overexpression significantly suppressed the activity of *hIL-6-3′UTR-WT* but not *hIL-6-3′UTR-Mut* (Figure 6I). These results indicate that RB upregulates hsa-miR-140 expression, and that the *hsa-mir-140-IL6* axis mediates RB function to suppress the self-renewal of human breast cancer cells.

## DISCUSSION

The data presented here demonstrate that Rb inactivation dysregulates an miRNA signature, causing significant changes in mRNA expression. These signatures were determined in the context of acquiring self-renewal activity in **p*53-*null mouse soft tissue sarcoma cells. We focused on *mir-140* in particular because of the significant expression change and conservation between human and mouse. Moreover, *hsa-mir-140* has been implicated in the suppression of a variety of human cancers, including breast cancer [16–20]. Combined with the information indicating the pivotal role of RB in the malignant progression of breast cancer [26–28], we anticipated a functional interaction between RB and *hsa-mir-140*. Previously, it was reported that *p*53, another well-known tumor suppressor, upregulates the transcription of tumor-suppressor miRNAs such as miR-34a/b/c/, miR-107, miR-145, miR-192, and miR-215, which regulate cell proliferation, apoptosis, and angiogenesis [29].

The induction of IL-6 secretion and subsequent autocrine/paracrine activation of STAT3 signaling supports the self-renewal activity of not only cancer cells [24, 30, 31] but also embryonic stem cells or induced pluripotent stem cells [32, 33]. The inactivation of RB functions is known to enhance stem cell-like activities [21, 34, 35]. This work proposes that the RB-miR-140-IL-6 axis may play a critical role in cancer stem cells. Our findings coincide with the recent finding that *hsa-mir-140* downregulation promotes cancer stem cell formation in breast cancer cells [20]. We however point out that miR-140 is not solely a mechanism that links RB to IL-6, since as compared to the mild effect of RB depletion on hsa-miR-140 expression in MCF-7 cells, IL-6 induction was more robust (Figure 6H and 6B). This suggests that multiple mechanisms are involved in RB-IL-6 linkage, which we are currently investing (Kitajima *et al*., unpublished).

A limitation of this study is that we did not determine how RB upregulates miR-140 expression. Compared to the negative regulation of target genes by RB, the mechanism of positive regulation by RB remains poorly understood. Both *mmu-mir-140* and *hsa-mir-140* are encoded in an intronic region of *WWP2* gene encoding an E3 ubiquitin ligase. The transcription of these *mir-140*s is positively regulated by SOX family members including SOX5, 6 and 9; these SOX genes are negatively regulated through DNA methylation in their transcriptional regulatory regions [20, 36–38]. We are currently testing the possibility that RB depletion promotes demethylation in their promoter regions.

In addition to *Il-6*, the genes induced by Rb depletion possibly in a *mir-140*-dependent manner included those encoding various secreted proteins such as proteases, growth factors, cytokines, and chemokines (Table 1). GO analysis identified an enrichment of genes that are typically involved in immune responses and extracellular activities (Figure 4A). Among secreted protein genes, the 3′UTR *of VEGF* gene can be targeted by hsa-miR-140 [21], and reportedly *VEGF* gene is induced by RB inactivation [39]. These facts may increase the authenticity of the genes listed in Figure 4B, which includes genes such as *Il-11, Hgf, Vegfa*, and *Wnt5a*, all of which are implicated in the malignant behavior of tumor cells [40–43]. Moreover, reportedly *mir-140* expression is implicated in inflammatory disease. Inflammatory cytokines, including IL-1β and TNF-α, downregulate *mir-140* expression leading to the development of osteoarthritis, most likely due to accelerated inflammation and/or ADAMTS5 activation [44]. Because RB is prevalently inactivated by various oncogenic signals during the malignant progression of many types of cancers, *mir-140* can potentially serve as a therapeutic tool for disrupting linkages of oncogenic signals to inflammatory responses, cell proliferation, or pro-angiogenic responses.

## MATERIALS AND METHODS

### Mice

**p*53*-knockout mice [45] were obtained from RIKEN BioResource Center (Ibaragi, Japan; #CDB0001K). C57BL/6 mice were purchased from Japan SLC. Animals were handled in accordance with the animal welfare guidelines of the Kanazawa University.

### Primary cell culture

Minced pieces of soft tissue sarcoma samples derived from *p53*-knockout mice were digested with 300 U/ml collagenase, 100 U/ml hyaluronidase and 100 μg/ml DNase I in α-modified Eagle's medium (αMEM) supplemented with 10% fetal bovine serum (FBS). The resulting cells were maintained in αMEM supplemented with 10% FBS.

### Sphere formation assay

Cells cultured under monolayer conditions were detached with trypsin-EDTA, and filtered through a 40 μm cell strainer. The cells were then inoculated into 1% methylcellulose-containing serum-free αMEM supplemented with B27 (Life Technologies, Carlsbad, USA), 10 ng/ml human EGF (PeproTech, Rocky Hill, USA) and 10 ng/ml human bFGF (PeproTech), at a density of 5 × 10^3^ cells per well on 6-well-type ultra-low attachment plate (EZ-BindShut II, AGC Techno Glass, Shizuoka, Japan). After 10–14 days incubation, spheres were observed with the assistance of an inverted phase contrast microscopy, and analyzed by BZ analysis software on a BZ-9000 fluorescence microscope (Keyence, Osaka, Japan) using the Hybrid Cell Counting module. Sphere-forming units were determined at a given day by counting cell aggregates with larger than 3,000 μm^2^ surface area and with the ratio of the longest diameter and the shortest diameter (L/S ratio) less than 1.5 (spherical figure). The whole area in a dish was scanned by automated microscope, and sphere number per dish was calculated from tiled image data.

### Establishment of secondary cells

More than 10 spheres induced by Rb depletion were manually picked up by micropippette under the microscope, collected into a 15 ml centrifuge tube, resuspended in 2 ml of 10% FBS-containing αMEM, disaggregated with cell a 40-μm strainer, and plated onto a 2D culture dish. To establish control secondary cells, all tumor cells in the sphere culture were collected into a 15 ml centrifuge tube after 2 weeks of culture, washed twice with 5 ml of serum-free αMEM, resuspended in 2 ml 10% FBS-containing αMEM, and then plated onto a 2D culture dish.

### Cell lines

NIH3T3 cells (gifted from Dr. Makoto Noda, Kyoto University) and MCF-7 cells (RIKEN BRC, RCB1904) were cultured in Dulbecco's modified Eagle's medium (DMEM) containing 10% FBS.

### Generation of lentivirus

MISSION TRC validated shRNA target sets for mouse *Rb* (#1:TRCN0000042543, #2: TRCN0000042544), human *RB* (#1TRCN0000040163, #2: TRCN0000010419) and negative control (SHC002) were purchased from Sigma-Aldrich (Missouri, USA). Murine microRNA precursor constructs for *mmu-mir-140* (MMIR-140-PA-1) and mouse precursor scramble negative control (MMIR-000-PA-1) were purchased from System Biosciences (Mountain View, USA). Generation and infection of lentivirus were performed according to the manufacturer's instruction. pQCXIH-PSM-RB7LP was purchased from Addgene (#37106). RB7LP lacking stop codon was amplified by PCR using primers attB1-7LP (GGGGACAAGTTTGTACAAAAAAGCAGGCTTCGCCACCATGAACACTATCCAACA) and attB2-7LP (GGGGACCACTTTGTACAAGAAAGCTGGGTTTTTCTCTTCCTTGTTTGAGGTATCCA). PCR products was cloned into pDONR223, sequenced by ABI 3130 sequencer, and then subcloned into pLenti6.3/V5-DEST.

### Quantitative RT-PCR (RT-qPCR)

Total RNA was extracted by using a miRNeasy Mini Kit (Qiagen). Reverse transcription was performed by using TaqMan MicroRNA Reverse Transcription Kit (Applied Biosystems, Foster City, USA), and real-time PCR quantitation was performed by using TaqMan Universal Master Mix II (Applied Biosystems) with LightCycler 480 System II (Roche, Basel, Switzerland), according to the manufacturer's instructions. To measure mRNA, total RNA was extracted by using TRIzol (#15596018, Life Technologies), reverse-transcribed using High Capacity RNA-to-cDNA Kit (Applied Biosystems), and quantitated using TaqMan Gene Expression Master Mix (Applied Biosystems), according to the manufacturer's instructions. The TaqMan probes used were mmu-miR-140, hsa-miR-140-3p, snoRNA202, RNU48, *Il6*, *IL6*, *Rb1*, *RB1*, *Actb* and *ACTB* (Applied Biosystems, assay ID 001187, 002234, 001232, 001006, Mm00446190_m1, Hs00985639_m1, Mm00485586_m1, Hs01078066_m1, Mm00607939_s1 and Hs99999903_m1). snoRNA202, RNU48, *Actb* and *ACTB* were used as internal controls.

### Immunoblotting (IB)

Whole-cell and nuclear fractions were prepared as described previously [46]. IB was conducted as described previously [46] using the following antibodies to: Phospho-Rb (#9308, Cell Signaling Technology), Total RB (#554136, BD Biosciences), Cyclin D1 (#2926, Cell Signaling Technology), α-Tubulin (#CP06, Calbiochem) and β-Actin (#3700, Cell Signaling Technology).

### RNA sequencing

The total RNA was extracted using the TRIzol reagent (#15596018, Life Technologies). From 15 μg of total RNA, a RNA-sequence library was constructed using the mRNA-sequence Sample Preparation Kit, according to the manufacturer's instructions (Illumina, California, USA). A HiSeq 2000 sequencer was used to generate 36-bp single-end-read RNA sequence tags according to the manufacturer›s protocol. The RNA-sequence tags were mapped to the mouse genomic sequence (mm 9 from the UCSC Genome Browser) using the ELAND program (Illumina). Unmapped or redundantly mapped sequences were removed from the dataset, and only uniquely mapped sequences without any mismatches were used for analyses [47]. The raw data of RNA sequence are available in DNA Data Bank of Japan (DDBJ) (DRA002910 and DRA002911).

### *In vivo* tumor formation assay

Cells were suspended (1 × 10^5^ cells/sample) in 50 μl αMEM with 10% FBS and 50 μl Matrigel (BD Biosciences), and then they were injected subcutaneously into C57BL/6 male mice. After 24 days, the mice were sacrificed for separating tumors, and tumors were separated and weighed.

### miRNA microarray

Total RNA was extracted by using the miRNeasy Mini Kit (Qiagen, Tokyo, Japan). The quality of the RNA samples was assessed using the Agilent 2100 Bioanalyzer 2100 (Agilent, Santa Clara, USA). The microRNA microarray analysis was performed with a Mouse miRNA microarray 8 × 15K Rel. 15.0 (Agilent, #29152). The fluorescence intensity was measured with a G2505B Micro Array Scanner (Agilent) in the Institute for Gene Research, Kanazawa University (Ishikawa, Japan). The data were normalized using the quantile method with GeneSpring 12.6.1 GX software (Agilent). The raw data are available in the Gene Expression Omnibus (GEO) (GSE77222).

### Determination of miRNAs regulated by Rb

miRNA microarray data were analyzed using one-way ANOVA of the three groups regarding 252 miRNAs, in which the false discovery rate (FDR) was controlled with the Benjamini-Hochberg (BH) procedure. The 37 miRNAs with an adjusted *p* value < 0.1 were subjected in succession to Dunnett's test using the control sample group as a control. Those miRNAs that had less than 0.05 of the adjusted *p* value in both comparisons of *Rb* shRNA vs. Non-target and *Rb* shRNA secondary vs. non-target, and where both comparisons of altered expression with in agreement (up- or down-regulation), were regarded as candidate miRNAs regulated by Rb.

### Generation of retroviruses

Retroviruses were recovered from the Platinum-E retroviral packaging cell line gifted from Dr. Toshio Kitamura (The Institute of Medical Science, The University of Tokyo). Platinum-E cells were maintained in DMEM supplemented with 10% FBS, and transfected with pMXs or pMXs- human RB. For retrovirus production, 6 × 10^6^ Platinum-E cells were transfected with 10 μg of each pMXs vector in 46 mg/ml polyethyleneimine. After 48 hours, the medium containing retrovirus particles was collected, passed through a 0.45 μm filter, and concentrated using polyethylene glycol.

### Determination of genes regulated by Rb

The tag count data from the RNA sequencing analysis were normalized by the iDEGES/edgeR method. Differentially Expressed Genes (DEGs) were estimated using the edgeR method to determine the genes that had an FDR threshold of 5% as DEGs. These analyses were computed in the ‘TCC’ package by using R 3.1.0 [48].

### Reporter assay

For the 3′UTR reporter assay, the 3′ UTR of mouse Il-6 and human IL-6 were amplified by PCR from mouse cDNA. The resulting fragment was inserted into the pmirGLO vector (Promega Corporation, Madison, USA), to generate a luciferase reporter construct *IL-6-3*′*UTR* according to the manufacturer's instructions. We also mutated complementary seed sequences in the miR-140-binding region (See Figure 4B and Supplementary Figure 4A), and generated the reporter construct *Il-6-3*′*UTR Mu*t. NIH3T3 cells were transfected with 0.5 μg reporter construct, either 3.0 μg mmu-miR-140 construct or 3.0 μg scramble control construct, and 0.25 μg β-galactosidase using 100 μl Opti-MEM (Life Technologies) and 11.25 μl FuGENE6 (Promega Corporation, Cat. #E2691) in 6-well-type plates according to the recommended protocol. At 48 hours post transfection, luciferase activity was assessed using 1 mM D-luciferin potassium salt. Luciferase activity was normalized using β-galactosidase. MCF-7 cells were transfected with 0.05 μg reporter construct and either 0.3 μg hsa-miR-140 construct or 0.3 μg scramble control construct using 10 μl Opti-MEM (Life Technologies, Cat. #31985-700) and 1.2 μl FuGENE6 in 96-well-type plates according to the recommended protocol. At 24 hours post transfection, luciferase activity was assessed using Dual-Glo Luciferase assay system (Promega Corporation, Cat. #E2920).

### Cap analysis gene expression (CAGE) sequencing

RNA was extracted using a miRNeasy Mini Kit (Qiagen). RNA quality was assessed by Bioanalyzer (Agilent) and standardized with an RNA integrity number (RIN) > 7.0. RNA purity was analyzed by Nano Drop and considered good-quality when A260/280 and A260/230 ratios were > 1.7. First strand cDNAs were transcribed to the 5′ end of capped RNAs, attached to CAGE “bar code” tags (Supplementary Table 2) and the sequenced CAGE tags were mapped to the mouse mm9 genomes using BWA software (v0.5.9) after discarding ribosomal or non-A/C/G/T base-containing RNAs. For tag clustering, CAGE-tag 5′ coordinates were input for Reclu clustering, with a maxmum irreproducible discovery rate of (IDR) 0.1 and minimum tags per million (TPM) value of 0.1 [49, 50]

### Determination of mir-140-dependent Rb inactivation signature

Genes were filtered into the Rb inactivation signature if their TPM were: 1) > 1.0 in scramble miRNA-overexpressed and Rb-depleted cells, and 2) > 1.5 times higher in either miRNA-overexpressed or Rb-depleted cells than in control cells. From that subset genes, genes with decreased expression in *mir-140*-overexpressed and RB-depleted cells compared with scramble miRNA-overexpressed and Rb-depleted cells were identified, 412 genes total.

### Statistical analysis

Statistical significance was assessed using unpaired two-tailed Student's *t-test* (indicated by horizontal and vertical bars) or one-way ANOVA followed by Tukey's post-hoc test (indicated by horizontal bars). *p* values less than 0.05 were considered significant as denoted by an asterisk (*). Data were presented as mean ± standard deviation (S.D.). In one-way ANOVA followed by Tukey's post-hoc tests, only pairs of our interest to the study were indicated.

## SUPPLEMENTARY MATERIALS FIGURES AND TABLES







## References

[1] Ha M, Kim VN (2014). Regulation of microRNA biogenesis. Nat Rev Mol Cell Biol.

[2] Winter J, Jung S, Keller S, Gregory RI, Diederichs S (2009). Many roads to maturity: microRNA biogenesis pathways and their regulation. Nat Cell Biol.

[3] Esquela-Kerscher A, Slack FJ (2006). Oncomirs - microRNAs with a role in cancer. Nat Rev Cancer.

[4] Lin S, Gregory RI (2015). MicroRNA biogenesis pathways in cancer. Nat Rev Cancer.

[5] Valastyan S, Weinberg RA (2009). MicroRNAs: Crucial multi-tasking components in the complex circuitry of tumor metastasis. Cell Cycle.

[6] Lu J, Getz G, Miska EA, Alvarez-Saavedra E, Lamb J, Peck D, Sweet-Cordero A, Ebert BL, Mak RH, Ferrando AA, Downing JR, Jacks T, Horvitz HR (2005). MicroRNA expression profiles classify human cancers. Nature.

[7] Thomson JM, Newman M, Parker JS, Morin-Kensicki EM, Wright T, Hammond SM (2006). Extensive post-transcriptional regulation of microRNAs and its implications for cancer. Genes Dev.

[8] Merritt WM, Lin YG, Han LY, Kamat AA, Spannuth WA, Schmandt R, Urbauer D, Pennacchio LA, Cheng JF, Nick AM, Deavers MT, Mourad-Zeidan A, Wang H (2008). Dicer, Drosha, and outcomes in patients with ovarian cancer. N Engl J Med.

[9] Lin RJ, Lin YC, Chen J, Kuo HH, Chen YY, Diccianni MB, London WB, Chang CH, Yu AL (2010). microRNA signature and expression of Dicer and Drosha can predict prognosis and delineate risk groups in neuroblastoma. Cancer Res.

[10] Khoshnaw SM, Rakha EA, Abdel-Fatah TM, Nolan CC, Hodi Z, Macmillan DR, Ellis IO, Green AR (2012). Loss of Dicer expression is associated with breast cancer progression and recurrence. Breast Cancer Res Treat.

[11] Kumar MS, Lu J, Mercer KL, Golub TR, Jacks T (2007). Impaired microRNA processing enhances cellular transformation and tumorigenesis. Nat Genet.

[12] Chan JA, Krichevsky AM, Kosik KS (2005). MicroRNA-21 is an antiapoptotic factor in human glioblastoma cells. Cancer Res.

[13] Gregory PA, Bracken CP, Bert AG, Goodall GJ (2008). MicroRNAs as regulators of epithelial-mesenchymal transition. Cell Cycle.

[14] Blandino G, Fazi F, Donzelli S, Kedmi M, Sas-Chen A, Muti P, Strano S, Yarden Y (2014). Tumor suppressor microRNAs: a novel non-coding alliance against cancer. FEBS Lett.

[15] Takahashi C, Sasaki N, Kitajima S (2012). Twists in views on RB functions in cellular signaling, metabolism and stem cells. Cancer Sci.

[16] Yang H, Fang F, Chang R, Yang L (2013). MicroRNA-140-5p suppresses tumor growth and metastasis by targeting transforming growth factor beta receptor 1 and fibroblast growth factor 9 in hepatocellular carcinoma. Hepatology.

[17] Yuan Y, Shen Y, Xue L, Fan H (2013). miR-140 suppresses tumor growth and metastasis of non-small cell lung cancer by targeting insulin-like growth factor 1 receptor. PLoS One.

[18] Zhang W, Zou C, Pan L, Xu Y, Qi W, Ma G, Hou Y, Jiang P (2015). MicroRNA-140–5p inhibits the progression of colorectal cancer by targeting VEGFA. Cell Physiol Biochem.

[19] Lan H, Chen W, He G, Yang S (2015). miR-140-5p inhibits ovarian cancer growth partially by repression of PDGFRA. Biomed Pharmacother.

[20] Li Q, Yao Y, Eades G, Liu Z, Zhang Y, Zhou Q (2014). Downregulation of miR-140 promotes cancer stem cell formation in basal-like early stage breast cancer. Oncogene.

[21] Kitajima S, Kohno S, Kondoh A, Sasaki N, Nishimoto Y, Li F, Abdallah Mohammed MS, Muranaka H, Nagatani N, Suzuki M, Kido Y, Takahashi C (2015). Undifferentiated State Induced by Rb-p53 Double Inactivation in Mouse Thyroid Neuroendocrine Cells and Embryonic Fibroblasts. Stem Cells.

[22] Salah M, Nishimoto Y, Kohno S, Kondoh A, Kitajima S, Muranaka H, Nishiuchi T, Ibrahim A, Yoshida A, Takahashi C (2015). An in vitro system to characterize prostate cancer progression identified signaling required for self-renewal. Mol Carcinog.

[23] Gauthier ML, Berman HK, Miller C, Kozakeiwicz K, Chew K, Moore D, Rabban J, Chen YY, Kerlikowske K, Tlsty TD (2007). Abrogated response to cellular stress identifies DCIS associated with subsequent tumor events and defines basal-like breast tumors. Cancer Cell.

[24] Marotta LL, Almendro V, Marusyk A, Shipitsin M, Schemme J, Walker SR, Bloushtain-Qimron N, Kim JJ, Choudhury SA, Maruyama R, Wu Z, Gonen M, Mulvey LA (2011). The JAK2/STAT3 signaling pathway is required for growth of CD44(+)CD24(−) stem cell-like breast cancer cells in human tumors. The Journal of clinical investigation.

[25] Knudsen ES, Wang JY (1997). Dual mechanisms for the inhibition of E2F binding to RB by cyclin-dependent kinase-mediated RB phosphorylation. Mol Cell Biol.

[26] Arima Y, Inoue Y, Shibata T, Hayashi H, Nagano O, Saya H, Taya Y (2008). Rb depletion results in deregulation of E-cadherin and induction of cellular phenotypic changes that are characteristic of the epithelial-to-mesenchymal transition. Cancer Res.

[27] Bosco EE, Wang Y, Xu H, Zilfou JT, Knudsen KE, Aronow BJ, Lowe SW, Knudsen ES (2007). The retinoblastoma tumor suppressor modifies the therapeutic response of breast cancer. The Journal of clinical investigation.

[28] Nik-Zainal S, Davies H, Staaf J, Ramakrishna M, Glodzik D, Zou X, Martincorena I, Alexandrov LB, Martin S, Wedge DC, Van Loo P, Ju YS, Smid M (2016). Landscape of somatic mutations in 560 breast cancer whole-genome sequences. Nature.

[29] Hermeking H (2012). MicroRNAs in the p53 network: micromanagement of tumour suppression. Nat Rev Cancer.

[30] Korkaya H, Kim GI, Davis A, Malik F, Henry NL, Ithimakin S, Quraishi AA, Tawakkol N, D'Angelo R, Paulson AK, Chung S, Luther T, Paholak HJ (2012). Activation of an IL6 inflammatory loop mediates trastuzumab resistance in HER2+ breast cancer by expanding the cancer stem cell population. Molecular cell.

[31] Sansone P, Storci G, Tavolari S, Guarnieri T, Giovannini C, Taffurelli M, Ceccarelli C, Santini D, Paterini P, Marcu KB, Chieco P, Bonafe M (2007). IL-6 triggers malignant features in mammospheres from human ductal breast carcinoma and normal mammary gland. The Journal of clinical investigation.

[32] Brady JJ, Li M, Suthram S, Jiang H, Wong WH, Blau HM (2013). Early role for IL-6 signalling during generation of induced pluripotent stem cells revealed by heterokaryon RNA-Seq. Nat Cell Biol.

[33] Takahashi K, Yamanaka S (2016). A decade of transcription factor-mediated reprogramming to pluripotency. Nat Rev Mol Cell Biol.

[34] Kareta MS, Gorges LL, Hafeez S, Benayoun BA, Marro S, Zmoos AF, Cecchini MJ, Spacek D, Batista LF, O'Brien M, Ng YH, Ang CE, Vaka D (2015). Inhibition of pluripotency networks by the rb tumor suppressor restricts reprogramming and tumorigenesis. Cell stem cell.

[35] Kohno S, Kitajima S, Sasaki N, Takahashi C (2016). Retinoblastoma tumor suppressor functions shared by stem cell and cancer cell strategies. World J Stem Cells.

[36] Yamashita S, Miyaki S, Kato Y, Yokoyama S, Sato T, Barrionuevo F, Akiyama H, Scherer G, Takada S, Asahara H (2012). L-Sox5 and Sox6 proteins enhance chondrogenic miR-140 microRNA expression by strengthening dimeric Sox9 activity. J Biol Chem.

[37] Nakamura Y, He X, Kato H, Wakitani S, Kobayashi T, Watanabe S, Iida A, Tahara H, Warman ML, Watanapokasin R, Postlethwait JH (2012). Sox9 is upstream of microRNA-140 in cartilage. Appl Biochem Biotechnol.

[38] Yang J, Qin S, Yi C, Ma G, Zhu H, Zhou W, Xiong Y, Zhu X, Wang Y, He L, Guo X (2011). MiR-140 is co-expressed with Wwp2-C transcript and activated by Sox9 to target Sp1 in maintaining the chondrocyte proliferation. FEBS Lett.

[39] Gabellini C, D Del Bufalo, Zupi G (2006). Involvement of RB gene family in tumor angiogenesis. Oncogene.

[40] Yu H, Lee H, Herrmann A, Buettner R, Jove R (2014). Revisiting STAT3 signalling in cancer: new and unexpected biological functions. Nat Rev Cancer.

[41] Gherardi E, Birchmeier W, Birchmeier C, Vande Woude G (2012). Targeting MET in cancer: rationale and progress. Nat Rev Cancer.

[42] Goel HL, Mercurio AM (2013). VEGF targets the tumour cell. Nat Rev Cancer.

[43] Anastas JN, Moon RT (2013). WNT signalling pathways as therapeutic targets in cancer. Nat Rev Cancer.

[44] Miyaki S, Asahara H (2012). Macro view of microRNA function in osteoarthritis. Nat Rev Rheumatol.

[45] Tsukada T, Tomooka Y, Takai S, Ueda Y, Nishikawa S, Yagi T, Tokunaga T, Takeda N, Suda Y, Abe S (1993). Enhanced proliferative potential in culture of cells from p53-deficient mice. Oncogene.

[46] Kitajima S, Miki T, Takegami Y, Kido Y, Noda M, Hara E, Shamma A, Takahashi C (2011). Reversion-inducing cysteine-rich protein with Kazal motifs interferes with epidermal growth factor receptor signaling. Oncogene.

[47] Kanai A, Suzuki K, Tanimoto K, Mizushima-Sugano J, Suzuki Y, Sugano S (2011). Characterization of STAT6 target genes in human B cells and lung epithelial cells. DNA research.

[48] Sun J, Nishiyama T, Shimizu K, Kadota K (2013). TCC: an R package for comparing tag count data with robust normalization strategies. BMC Bioinformatics.

[49] Murata M, Nishiyori-Sueki H, Kojima-Ishiyama M, Carninci P, Hayashizaki Y, Itoh M (2014). Detecting expressed genes using CAGE. Methods Mol Biol.

[50] Ohmiya H, Vitezic M, Frith MC, Itoh M, Carninci P, Forrest AR, Hayashizaki Y, Lassmann T, Consortium F (2014). RECLU: a pipeline to discover reproducible transcriptional start sites and their alternative regulation using capped analysis of gene expression (CAGE). BMC Genomics.

[51] Betel D, Koppal A, Agius P, Sander C, Leslie C (2010). Comprehensive modeling of microRNA targets predicts functional non-conserved and non-canonical sites. Genome Biol.

[52] Betel D, Wilson M, Gabow A, Marks DS, Sander C (2008). The microRNA.org resource: targets and expression. Nucleic Acids Res.

