# Supplementary material for: MicroRNA-140 mediates RB tumor suppressor function to control stem cell-like activity through interleukin-6
Source: Oncotarget. 2017 Jan 16;8(8):13872–85. doi: 10.18632/oncotarget.14681 (PMC5355146; doi:10.18632/oncotarget.14681)
Supplement: Supplementary file 1 [file oncotarget-08-13872-s001.pdf]

MicroRNA-140 mediates RB tumor suppressor function to control stem cell-like activity through interleukin-6

Supplementary Materials

BrdU incorporation

2×10^5 cells were incubated with 10 µM BrdU at 37°C for 20 min in αMEM supplemented 10% FBS. BrdU staining was performed according to the manufacturer's instruction (#11 296 736 001, Roche). 30,000 cells suspended in 500 µl of PBS containing 5% FBS were analyzed by FACSCanto (BD Biosciences).

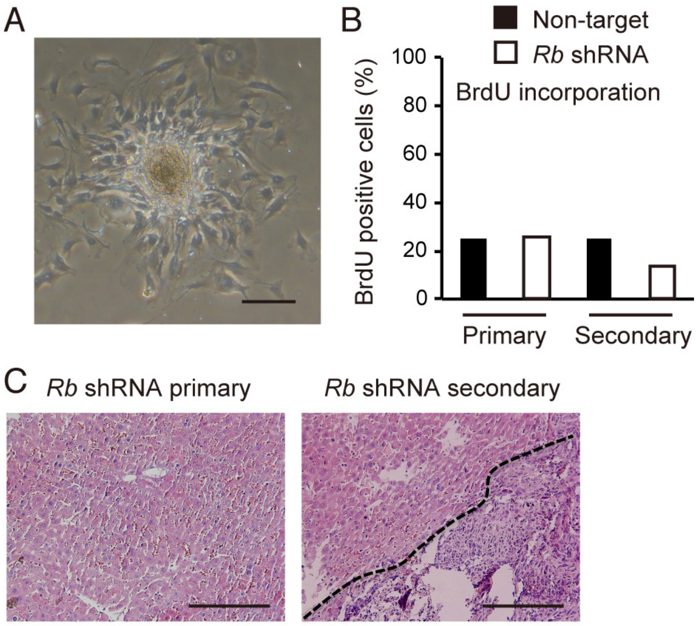

Supplementary Figure 1: Analysis of spherogenic cells induced by Rb depletion. (**A**) A phase-contrast image of cells migrating from a sphere that had been generated under sphere culture condition, relocated onto a new culture dish, and grown under 2D culture condition. (**B**) Percentage of BrdU positive cells determined by FACS in *p53*-null soft tissue sarcoma cells transduced with the indicated shRNA and cultured under the indicated condition. $N = 1$. (**C**) Hematoxylin-Eosin (HE) staining of the liver of C57BL/6 mice those were intra-abdominally inoculated with 1×10^4 of the indicated cells. (day 29). Scale bar: 100 µm. The right lower part of the right Figure indicated by dotted line shows metastasis.

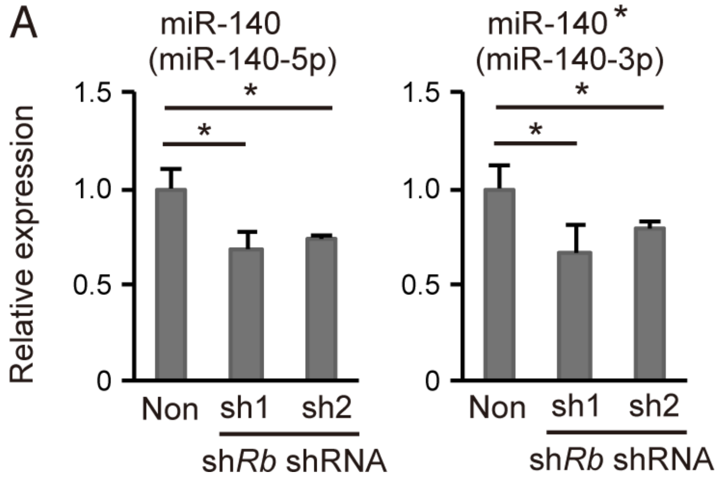

Supplementary Figure 2: Downregulation of *mmu-mir-140* expression following Rb depletion. (**A**) RT-qPCR of *miR-140* (left) and *miR-140** (right) in *p53*-null soft tissue sarcoma cells transduced with the indicated vector. $N = 3$.

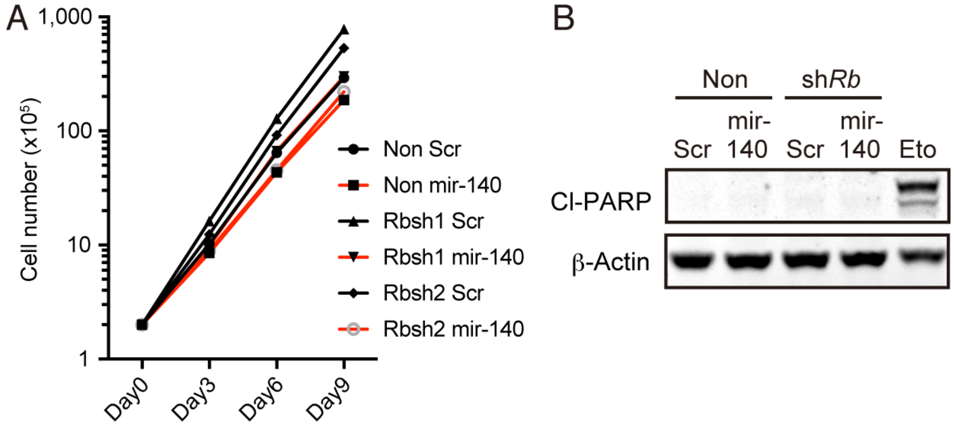

Supplementary Figure 3: Analysis of cell growth and apoptosis in *mmu-mir-140 expressing* p53-null soft tissue sarcoma cells. (**A**) Growth curve of *p53*-null soft tissue sarcoma cells transduced with the indicated vector. (**B**) Immunoblot (IB) of the indicated proteins in *p53*-null soft tissue sarcoma cells transduced with the indicated vector. As a positive control for apoptosis induction, cells were treated with 5 μM etoposide for 48 h.

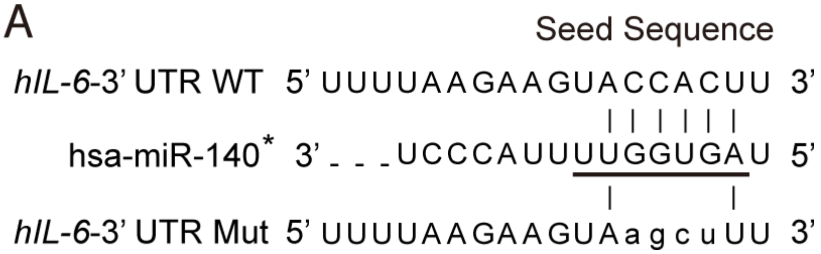

Supplementary Figure 4: The sequence of the reporter constructs *hIl-6-3'UTR WT* and *hIl-6-3'UTR Mut*. (**A**) The sequence of the human *IL-6 3'UTR* containing seed sequence of *hsa-mir-140* (underlined). The seed sequence of the human *IL-6 3'UTR* was mutated as shown (*hIL-6-3'UTR Mut*).

Supplementary Table 1: The list of 412 genes induced by Rb depletion possibly in a mir140-dependent manner in *p53*-null soft tissue sarcoma cells (See Material and Methods). Gene list was ranked according to the average of tags per million (TPM) in scramble miRNA overexpressed and Rb depleted cells determined by cap analysis gene expression (CAGE) sequencing ($N = 3$). See Supplementary_Table_1

Supplementary Table 2: Gene ontology (GO) analysis using the 412 genes listed in Table S1. The GO terms with p value < 0.05 are listed. See Supplementary_Table_2
